# Cerebrospinal Fluid Concentration of Brain-Derived Neurotrophic Factor and Cognitive Function in Non-Demented Subjects

**DOI:** 10.1371/journal.pone.0005424

**Published:** 2009-05-01

**Authors:** Ge Li, Elaine R. Peskind, Steven P. Millard, Peter Chi, Izabela Sokal, Chang-En Yu, Lynn M. Bekris, Murray A. Raskind, Douglas R. Galasko, Thomas J. Montine

**Affiliations:** 1 Department of Psychiatry and Behavioral Sciences, University of Washington School of Medicine, Seattle, Washington, United States of America; 2 Department of Pathology, University of Washington School of Medicine, Seattle, Washington, United States of America; 3 Department of Medicine, University of Washington School of Medicine, Seattle, Washington, United States of America; 4 Mental Illness Research, Education, and Clinical Center, Veterans Affairs Puget Sound Health Care System, Seattle, Washington, United States of America; 5 Geriatric Research, Education, and Clinical Center, Veterans Affairs Puget Sound Health Care System, Seattle, Washington, United States of America; 6 Department of Neurosciences, University of California San Diego, La Jolla, California, United States of America; James Cook University, Australia

## Abstract

**Background:**

Brain-derived neurotrophic factor (BDNF) is an activity-dependent secreted protein that is critical to organization of neuronal networks and synaptic plasticity, especially in the hippocampus. We tested hypothesis that reduced CSF BDNF is associated with age-related cognitive decline.

**Methodology/Principal Findings, and Conclusions/Significance:**

CSF concentration of BDNF, Aβ_42_ and total tau were measured in 128 cognitively normal adults (Normals), 21 patients with Alzheimer's disease (AD), and nine patients with Mild Cognitive Impairment. *Apolipoprotein E* and *BDNF* SNP *rs6265* genotype were determined. Neuropsychological tests were performed at baseline for all subjects and at follow-up visits in 50 Normals. CSF BDNF level was lower in AD patients compared to age-matched Normals (p = 0.02). CSF BDNF concentration decreased with age among Normals and was higher in women than men (both p<0.001). After adjusting for age, gender, education, CSF Aβ_42_ and total tau, and *APOE* and *BDNF* genotypes, lower CSF BDNF concentration was associated poorer immediate and delayed recall at baseline (both p<0.05) and in follow up of approximately 3 years duration (both p<0.01).

**Conclusions/Significance:**

Reduced CSF BDNF was associated with age-related cognitive decline, suggesting a potential mechanism that may contribute in part to cognitive decline in older individuals.

## Introduction

Age-related cognitive decline is a complex convergent phenotype that likely derives in part from brain senescence and in part from prodromal dementing illnesses, most commonly Alzheimer's disease (AD). Brain-derived neurotrophic factor (BDNF) is an activity-dependent secreted protein that, along with its receptors, is expressed widely in the central nervous system and is critical to organization of neuronal networks and synaptic plasticity, especially in the hippocampus, in a variety of animal models and apparently in humans [1], [2]. Although controversy remains, these human data derive mostly from investigations of an allelic variant of BDNF (*Val66Met* or *Met-BDNF*), inheritance of which has been associated with poorer cognitive performance in healthy older adults [3], impaired memory in patients with schizophrenia and healthy controls [1], [2], abnormal hippocampal activation as assessed by fMRI [2], and an approximately 4% to 11% smaller hippocampal volume as determined by MRI in healthy adult volunteers [3], [4]. The mechanisms that underlie these associations of functional and structural differences with inheritance of *Met-BDNF* are not clear; however, one study has shown diminished depolarization-induced secretion of *Met-BDNF* compared to *Val-BDNF*, and failure of *Met-BDNF* to localize to secretory granules or synapses in transfected neurons [2]. Together, these findings have led to the hypothesis that reduced BDNF secretion is one mechanism of age-related cognitive decline. We are unaware of any published study that has yet tested this hypothesis.

## Methods

All procedures were approved by the institutional review boards of the University of Washington; all subjects were recruited from University of Washington Alzheimer's Disease Research Center and provided written informed consent. Subjects underwent detailed clinical and laboratory evaluation and were classified as no cognitive impairment (Normals), amnestic mild cognitive impairment (MCI) [5], or probable AD [6]. Inclusion and exclusion criteria, method of CSF collection, and analysis were exactly as previously described [7].

CSF concentration of BDNF, total tau and Aβ_42_ was measured using an X-MAP-based assay [7]. *APOE* genotype was determined by a restriction digest method [8]. *BDNF* SNP *rs6265* was genotyped using TaqMan allelic discrimination detection, as previously described [9].

Neuropsychological tests included: (i) Paragraph Recall - a test of declarative memory [10], [11]. Paragraphs were modeled after the Logical Memory subtests I and II of the WMS-R [12]. Total score for immediate recall and delayed recall (each with possible range 0–25) were used. (ii) Category Fluency - a test of semantic memory [13]. Total number of unique animals named in 60 seconds was used. (iii) Trail Making Test, Parts A and B - test of ability to adapt to shifting task demands. Time taken to complete Part B (upper bound of 300 sec), a measure of executive function [14], was used.

One-way analysis of variance (ANOVA) was used with *post hoc* Bonferroni multiple pair-wise comparisons for continuous variables and Fisher's exact test for categorical variables to assess differences between diagnostic and genetic groups. Linear regression models were used for associations of demographic characteristics with CSF BDNF concentration, and cross-sectional relationships between CSF BDNF concentration and coincident cognitive test performance. Raw scores were used for each test except log-transformed times for Trails B to remove skewness. We used two-stage regression (least squares slope for each test in each individual over time, then weighted regression model with slope as response variable) to assess association of baseline CSF BDNF concentration with subsequent longitudinal changes of cognitive test performance [15]. Weights were based on subjects having different numbers of follow-up visits at different times after baseline. Statistical analyses were performed using S-PLUS version 8.0 [16] and R version 2.7.1 [17].

## Results

### Age-matched Normals, MCI, and AD

**Table 1** presents demographics and baseline CSF BDNF levels in 128 normal controls (Normals), 9 MCI and 21 AD patients. To compare measures between these three groups after adjusting for age, a subset of the Normals (n = 76) age≥50 years (Older Normals, ON) was used as a comparison group. CSF BDNF level was lower in AD patients compared to ON with a mean difference of 25 pg/ml (*post-hoc* Bonferroni test, p = 0.02). CSF BDNF level for the nine MCI patients was not different from either group (both p>0.05). The frequencies of *Met-BDNF* genotype (*G/G vs. G/A or A/A*) were not different between ON, MCI, and AD subjects (Fisher's Exact Test p = 0.36). Moreover, CSF BDNF concentration in AD subjects was not different among *Met-BDNF* genotype *G/G* (70%, 217±38 pg/ml), *G/A* (25%, 214±39 pg/ml), or *A/A* (5%, 213 pg/ml, n = 1) (F = 0.02, df = 2, p = 0.99).

**Table 1:** Subject characteristics and CSF BDNF levels at baseline.

	Normals	Normals With Follow-up	ON (Normals age≥50)	MCI	AD	p-value (ON, MCI & AD)**
**Number**	128	50	76	9	21	
**Age, years, mean±SD (Range)**	52±20 (21–100)	72±9 (41–100)	67±10 (50–100)	74±8 (63–82)	68±10 (52–87)	0.14
**Gender, male %**	49	46	43	67	48	0.45
**Race, Caucasian %** *	90	90	89	100	95	0.29
***APOE*** ** Genotype, any ** ***ε4*** ** %** *	30	28	35	78	65	0.004
***BDNF*** ** Genotype ** ***rs6265 G/A*** ** or ** ***A/A*** ** %** *	40	34	39	12	30	0.36
**Education, years mean±SD** *	16±3	16±3	16±3	16±3	15±3	0.33
**BDNF, pg/ml, mean±SD**	246±33	233±35	240±36	241±36	216±35	0.02
**MMSE, mean±SD**	29±1	29±1	29±1	28±1	20±5	

**ON**: Older Normals; **MCI**: Mild Cognitive Impairment; **AD**: Alzheimer disease; MMSE: Mini-Mental State Exam.

* Missing Values. Race – 1 (AD). APOE – 2 (one ON, one AD). BDNF Genotype – 4 (two ON, one MCI, one AD). Education – 1 (AD).

** p-value based on one-way ANOVA for continuous variables and Fisher's exact test for categorical variables. No comparison between groups was performed for MMSE since this test is used for diagnosis.

### CSF BDNF concentration in all cognitively normal subjects

Our focus was on 128 cognitively normal volunteers (Normals) in whom we measured CSF BDNF, Aβ_42_, and total tau, and who underwent neuropsychological evaluation (**Table 1**). CSF BDNF concentration was not different among *rs6265* genotype in Normals: *G/G* (60%, 242±37 pg/ml), *G/A* (36%, 253±26 pg/ml), or *A/A* (5%, 251±28 pg/ml) (F = 1.52, df = 2, p = 0.22).

**Figure 1:**
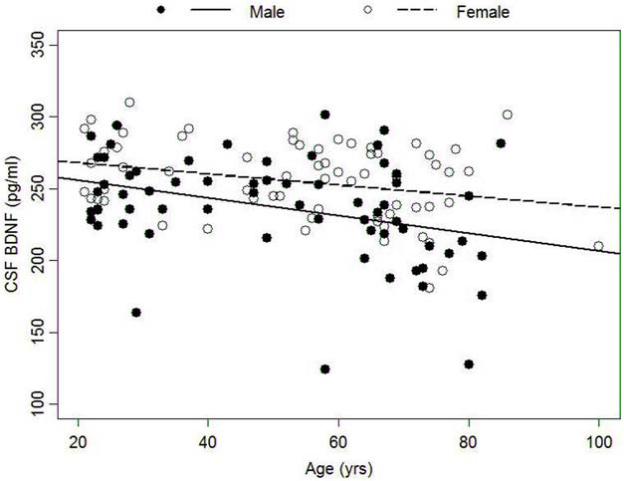
Cross-sectional relationships between age and CSF BDNF concentration by gender.

CSF BDNF concentration decreased with age among Normals and was higher in women than men (both p<0.001; **Figure 1**). In a multivariate linear regression with CSF BDNF as the dependent variable and age, gender, presence of the *APOE* ε4 allele, and *Met-BDNF (G/G vs G/A or A/A)* as independent predictors, both age and gender were significantly associated with CSF BDNF level (p<0.001). Neither presence of *APOE ε4* allele (p = 0.33) nor *Met-BDNF* (p = 0.09) was related to CSF BDNF levels. In addition, there was no interaction between *APOE ε4* allele and *Met-BDNF* genotype (p = 0.25), nor between age and gender (p = 0.45). Removing the three lowest values of BDNF for males did not change the results. Finally, after adjusting for age and gender, CSF BDNF levels were related inversely (slope = −0.03) to CSF levels of Aβ_42_ (p = 0.05) but not to CSF total tau (p = 0.34).

#### Association between CSF BDNF concentration, memory, and cognitive ability in cognitively normal subjects at baseline

Most (n = 121) Normals underwent extensive neuropsychological testing at baseline (**Table 1**). **Table 2** shows the regression coefficients and p-values associated with baseline CSF BDNF levels for each cognitive test in multivariate regression models after adjusting for age, gender, and years of education (Model 1); adjusting for the previous variables as well as concentration of CSF Aβ_42_ and total tau (biomarkers of latent and prodromal AD [18], [19], Model 2); and adjusting for the previous variables as well as presence of *APOE ε4* and *Met-BDNF* (Model 3). There was an association between higher CSF BDNF concentrations and better performance on Paragraph Recall - Delayed that was independent of CSF Aβ_42_ and total tau concentrations (Model 2) as well as presence of *APOE ε4* and *Met-BDNF* (Model 3); correlation with the Paragraph Recall - Immediate was significant when all predictor variables were in the model (Model 3). An association between lower CSF BDNF and poor performance on Trail Making Test Part B also was observed. There was no significant correlation between CSF BDNF concentration and performance on the Category Fluency test.

**Table 2:** Relationships between baseline CSF BDNF concentration with cross-sectional and longitudinal cognitive performance.

	Cross-sectional (n = 121)			Longitudinal (n = 50)		
	Model 1	Model 2	Model 3	Model 4	Model 5	Model 6
**Paragraph Recall: Immediate Recall**	0.17 (0.11); 0.13	0.22 (0.12); 0.06	0.26 (0.12); 0.03	0.09 (0.04); 0.02	0.11 (0.04); 0.01	0.13 (0.04); <0.01
**Paragraph Recall: Delayed Recall**	0.32 (0.11); <0.01	0.39 (0.11); <0.01	0.43 (0.10); <0.01	0.13 (0.05); <0.01	0.13 (0.05); 0.01	0.15 (0.05); <0.01
**Category Fluency: Animal**	−0.08 (0.19); 0.67	0.02 (0.19); 0.93	0.06 (0.19); 0.73	0.16 (0.06); 0.01	0.19 (0.06); <0.01	0.21 (0.07); <0.01
**Log10 Trail Making test: Part B**	−0.009 (0.005); 0.07	−0.013 (0.005); 0.02	−0.011 (0.005); 0.03	−0.001 (0.001); 0.42	−0.002 (0.001); 0.14	−0.002 (0.001); 0.11

Data are: linear regression model coefficient (SE) per10 pg/ml; p-value. **Models 1, 2, and 3**: Cross-sectional relationships between baseline CSF BDNF concentration and coincident cognitive test scores in 121 Normals with baseline neuropsychological testing. Model 1: adjusted for age, gender, and years of education. Model 2: Model 1 plus CSF Aβ_42_ and total tau concentrations. Model 3: Model 2 plus *APOE ε*4 (*ε4*− vs. *ε4*+) and *Met-BDNF* genotype (*G/G* vs. *G/A* or *A/A*) (n = 119 because of two missing values for *rs6265* genotype). **Models 4, 5 and 6**: longitudinal relationships between baseline CSF BDNF concentration and subsequent annual change in cognitive test scores for 50 Normals with follow-up evaluation. Model 4: adjusted for age, gender, years of education, and baseline test score. Model 5: Model 4 plus baseline CSF Aβ_42_ and total tau concentrations. Model 6: Model 5 plus *APOE ε*4 (*ε*4− vs. *ε*4+) and *Met-BDNF* genotype (*G/G* vs. *G/A* or *A/A*).

#### Association between baseline CSF BDNF concentration and longitudinal changes in cognitive performance

**Figure 2:**
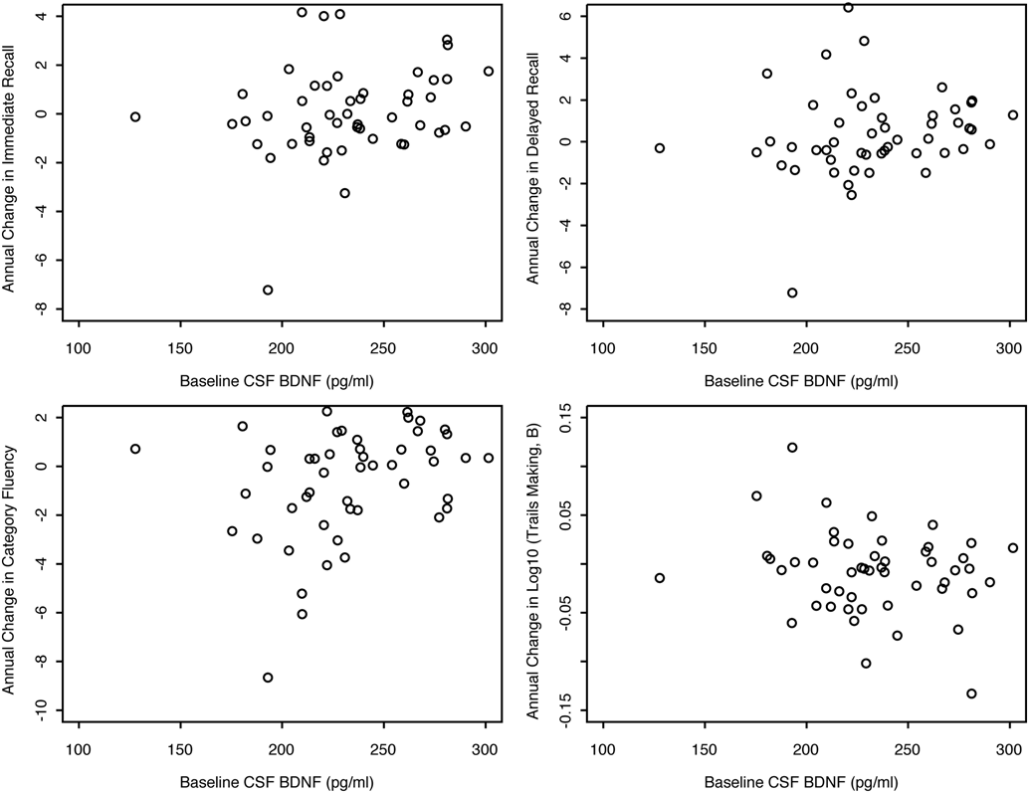
Annual change in cognitive test scores versus baseline CSF BDNF concentration.

Fifty Normals (27 women), aged 40–100 years (mean±SD = 71.5±8.9) had at least one clinical follow-up visit (mean±SD 4.0±1.1, Range = 2–6) with an average length of follow-up of 3.3 years (SD = 1.2 years, Range = 0.7–5.8 years) (**Table 1**). **Figure 2** shows unadjusted annual changes in cognitive test scores versus baseline CSF BDNF concentration. After adjusting for age, gender, years of education, and baseline test score, lower values of baseline BDNF concentration were significantly associated with greater annual decline in Immediate and Delayed Recall scores on the Paragraph Recall test, and a greater annual decline in the Category Fluency test (Model 4). These relationships were unchanged after further adjusting for baseline CSF Aβ_42_ and total tau concentrations (Model 5), suggesting that prediction by baseline CSF BDNF concentration for subsequent cognitive changes is independent of preclinical AD. These relationships also were unchanged after further adjusting for presence of *APOE ε4* and *Met-BDNF* (Model 6). All results were unaffected by removing the subject who had the largest annual decline for Immediate Recall, Delayed Recall, and Category Fluency (**Figure 2**).

### Relationship between BDNF genotype and cognitive performance in the Normals

Among all Normals (n = 119; 2 missing values for genotype), baseline performance on each of the four cognitive tests (Immediate and Delayed Recall, Category Fluency, and Trail Making Part B) did not differ by *Met-BDNF* genotype (*G/G vs. G/A or A/A*, all p>0.05), even after adjusting for age, gender and education in the multiple regression model (all p>0.05). Similarly, the longitudinal changes in these cognitive performances (n = 50) were not associated with *Met-BDNF* genotype (all p>0.05).

## Discussion

We made three novel observations in cognitively normal individuals. (i) CSF BDNF decreased across the human life-span in the absence of dementia or MCI and was independent of inheritance of *Met-BDNF* or the *APOE ε4* allele. (ii) Women had higher average CSF BDNF concentrations than men. (iii) Lower CSF BDNF concentration was associated strongly with poorer memory and less so with diminished executive function; importantly, these associations were independent of CSF biomarkers of preclinical AD, suggesting that the mechanisms that contribute to early AD and to age-related decline in CSF BDNF might be independent. We must acknowledge that this study has a relatively small sample size and short duration of follow-up and further studies are needed to validate our results.

While ours is the first study of which we are aware to investigate age-related changes in CSF BDNF, other studies have investigated disease-associated changes in CSF BDNF in patients with idiopathic Parkinson disease [7], [20] or in patients with AD [7], [21], [22]. We confirmed that CSF BDNF is further reduced beyond age-related decline in patients with probable AD. CSF and serum BDNF concentrations do not correlate [23]; yet, increased and decreased serum BDNF levels are related to early and late stages, respectively, of AD [24].

The gender difference in CSF BDNF observed in this study is especially interesting, and we speculate that this may be due to hormonal effects. Animal studies have shown that estrogen receptors colocalize to cells that express BDNF and its receptor trkB, and estrogen regulates the expression of BDNF [25]. The relationship between estrogen levels and BDNF expression and secretion, and their potential effect on the cognition in humans requires further studied.

Our data showed that progressive decline in CSF BDNF concentration was a feature of advancing age independent of preclinical AD or dementia, and was associated strongly with reduced performance in declarative memory tests and less strongly with performance on tests of executive function, perhaps a reflection of the special role of BDNF in hippocampal function. Age-related reduction in CSF BDNF was independent of inheritance of *Met-BDNF* or *APOE ε4*. Further reduction in CSF BDNF occurred in AD. Our data suggest that reduced secretion of BDNF in the central nervous system is one mechanism that may contribute to age-related cognitive decline.
